# Disulfiram/copper selectively eradicates AML leukemia stem cells *in vitro* and *in vivo* by simultaneous induction of ROS-JNK and inhibition of NF-*κ*B and Nrf2

**DOI:** 10.1038/cddis.2017.176

**Published:** 2017-05-18

**Authors:** Bing Xu, Shiyun Wang, Rongwei Li, Kai Chen, Lingli He, Manman Deng, Vinodh Kannappan, Jie Zha, Huijuan Dong, Weiguang Wang

**Affiliations:** 1 Department of Hematology, The First Affiliated Hospital of Xiamen University, Xiamen, China; 2 Department of Hematology, Nanfang Hospital, Southern Medical University, Guangzhou, China; 3 Research Institute in Healthcare Science, Faculty of Science and Engineering, University of Wolverhampton, Wolverhampton, UK

## Abstract

Acute myeloid leukemia (AML) is a heterogeneous malignancy. Despite the advances in past decades, the clinical outcomes of AML patients remain poor. Leukemia stem cells (LSCs) is the major cause of the recurrence of AML even after aggressive treatment making, promoting development of LSC-targeted agents is an urgent clinical need. Although the antitumor activity of disulfiram (DS), an approved anti-alcoholism drug, has been demonstrated in multiple types of tumors including hematological malignancies such as AML, it remains unknown whether this agent would also be able to target cancer stem cells like LSCs. Here, we report the *in vitro* and *in vivo* activity of DS in combination with copper (Cu) against CD34^+^/CD38^+^ leukemia stem-like cells sorted from KG1*α* and Kasumi-1 AML cell lines, as well as primary CD34^+^ AML samples. DS plus Cu (DS/Cu) displayed marked inhibition of proliferation, induction of apoptosis, and suppression of colony formation in cultured AML cells while sparing the normal counterparts. DS/Cu also significantly inhibited the growth of human CD34^+^/CD38^+^ leukemic cell-derived xenografts in NOD/SCID mice. Mechanistically, DS/Cu-induced cytotoxicity was closely associated with activation of the stress-related ROS-JNK pathway as well as simultaneous inactivation of the pro-survival Nrf2 and nuclear factor-*κ*B pathways. In summary, our findings indicate that DS/Cu selectively targets leukemia stem-like cells both *in vitro* and *in vivo*, thus suggesting a promising LSC-targeted activity of this repurposed agent for treatment of relapsed and refractory AML.

It is widely accepted that acute myeloid leukemia (AML), a clinically and biologically clonal disorder of hematopoietic progenitor cells, is initiated from a small subset of leukemia stem cells (LSCs).^1, 2^ LSCs are relatively quiescent and thus highly resistant to chemotherapy and radiotherapy, therefore primarily contributing to the dismal clinical outcome for AML.^3, 4^ Despite the conventional multi-drug combinational therapies, the most preferred and efficacious treatment, have largely improved the long-term disease-free survival (DFS) of patients with AML, virtually all of these patients will however eventually relapse owing to drug-resistant LSCs that are not eradicated by current standard therapies. Thus, this has become a major hindrance to success of AML treatment.^5^ To prolong the duration of DFS as well as overall survival, new therapeutic agents targeting LSCs with low/non systemic toxicity are urgently needed for AML treatment.

LSCs are well accepted to be a CD34-positive and CD38-negative cell population with the ability to reconstitute human AML in immunodeficient mice regardless of the morphological subtype of AML,^6^ although recent studies have suggested that LSCs could also exist in CD34^+^/CD38^+^ or CD34^−^ blast populations at least in some types of AML.^7^ KG1*α* and Kasumi-1 cell lines derived from male AML patients, both of which have high percentage of CD34^+^CD38^−^ population, are widely used for *in vitro* and *in vivo* studies of LSCs.^8^

Disulfiram (DS) is a Food and Drug Administration (FDA)-approved anti-alcoholism drug that has been used in clinic for >60 years.^9, 10^ As a divalent metal ion chelator, DS is able to strongly chelate copper (Cu) to form a disulfiram/copper (DS/Cu) complex that has been reported to be highly active against various types of tumors, including melanoma,^11, 12, 13^ breast cancer,^14, 15, 16^ colon cancer,^17^ prostate cancer,^18^ as well as hematological malignancies including myeloid leukemia,^19, 20^ but display low toxicity. However, it remains unknown whether DS/Cu would also be capable to target cancer stem cells such as LSCs.

Reactive oxygen species (ROS), the product of mitochondria oxidative phosphorylation, has a crucial role as an intracellular messenger in numerous biological events, including cell proliferation and survival. It is a consensus that excessive production of ROS results in peroxidation of lipid, protein, and DNA, leading to cellular damage and apoptosis.^21^ As tumor cells usually have to deal with higher levels of ROS than their normal counterparts, further increase of ROS by ROS-inducing agents, such as DS/Cu, could exhaust the cellular antioxidants, therefore resulting in apoptosis of tumor cells.^19, 22^ C-jun NH_2_-terminal kinase (JNK), an important member of the MAPK family, has a crucial role in a variety of stress-triggered responses, including differentiation and apoptosis.^23, 24^ Furthermore, it has also been demonstrated that ROS-mediated apoptosis is closely associated with persistent activation of the JNK pathway.^25, 26^

Nuclear factor-*κ*B (NF-*κ*B), a transcription factor usually activated by inflammatory stimuli and cellular stresses, has a pivotal role in regulation of cell survival and growth.^27^ NF-*κ*B has also been found to be essential for survival of cancer stem cells including LSCs, whereas inhibition of NF-*κ*B triggers apoptosis of these cells.^27^ NF-E2-related factor-2 (Nrf2) is a transcription factor that mediates cytoprotective antioxidant responses and thus prevents cells from the damage induced by ROS and other harmful chemicals in various types of cancers.^28^ In addition to its tumorigenic function through stress responses, activation of Nrf2 is also associated with drug resistance to standard chemotherapy as well as with poor survival of cancer patients.^29, 30, 31^ Therefore, a possibility arises whether simultaneous inhibition of NF-*κ*B and Nrf2 might lead to increased efficacy of an antitumor agent, thereby representing an attractive and promising avenue to success of cancer treatment, particularly due to targeting these two signals crucial for survival of cancer stem cells like LSCs.

Here, we report that DS in combination with Cu has a potent and selective anti-leukemia property *in vitro* against leukemia stem-like cells (e.g., CD34^+^/CD38^−^ KG1*α* and Kasumi-1 cells and primary CD34^+^ cells isolated from AML patients) as well as is highly effective *in vivo* in CD34^+^/CD38^−^ leukemic cell-derived xenograft mouse models, in association with induction of apoptosis via activation of the stress-related ROS-JNK pathway and inhibition of the pro-survival Nrf2 and NF-*κ*B pathways.

## Results

### Enrichment of leukemia stem-like cells from the human AML KG1*α* cell line

**Figure 1:**
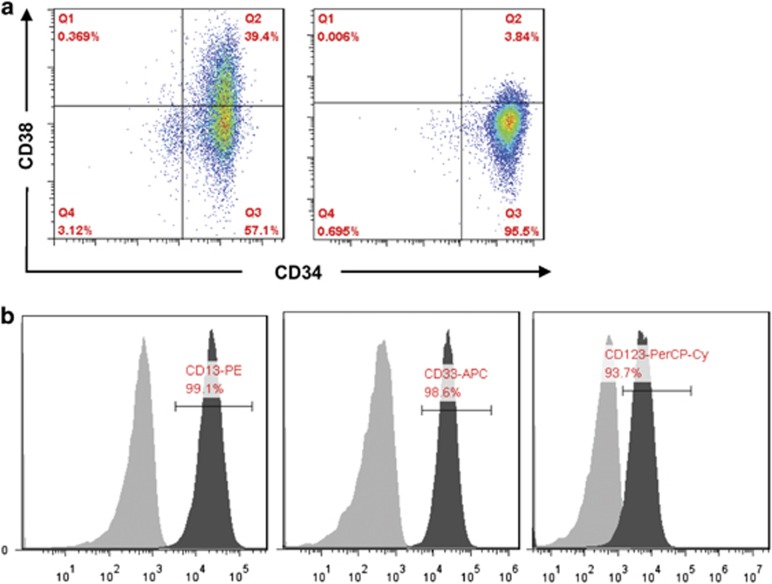
Enrichment of leukemia stem-like cells from KG1*α* cell line. Percentage of CD34^+^/CD38^−^ population was analyzed by flow cytometry before (**a**, left panel) and after sorting (right panel). Before sorting, the CD34^+^/CD38^−^ KG1a cells were 59.4±6.2%. After sorting, the percentage of CD34^+^/CD38^−^ is 93.2±2.7%. (**b**) Myeloid surface markers (CD13, CD33, and CD123) in sorted KG1a cells were detected by flow cytometry. The filled grey area represents isotype control staining

Leukemia stem-like cells were enriched from KG1*α* cell line, a subclone cell line of KG1 cells, by sorting a CD34^+^/CD38^−^ cell population using fluorescence-activated cell sorting (FACS). As shown in Figure 1a, percentage of the CD34^+^/CD38^−^ population was significantly increased after sorted from KG1*α* cells (93.2±2.7% *versus* 59.4±6.2% for KG1*α* cells before sorting; Figure 1a, right panel; *t*=−8.675, *P*=0.001). As KG1a cells might have lost myeloid features (e.g., the pan-myeloid antigen MY9),^32^ we further performed flow cytometry to monitor the markers of myeloid cells. As shown in Figure 1b, these CD34^+^/CD38^−^ leukemia stem-like cells displayed positivity of the myeloid markers CD13 (99.1%), CD33 (98.6%), and CD123 (93.7%), suggesting that certain myeloid features remain in these sorted CD34^+^/CD38^−^ KG1*α* cells.

### DS/Cu is cytotoxic against leukemia stem-like cells *in vitro* in a dose-dependent manner

**Figure 2:**
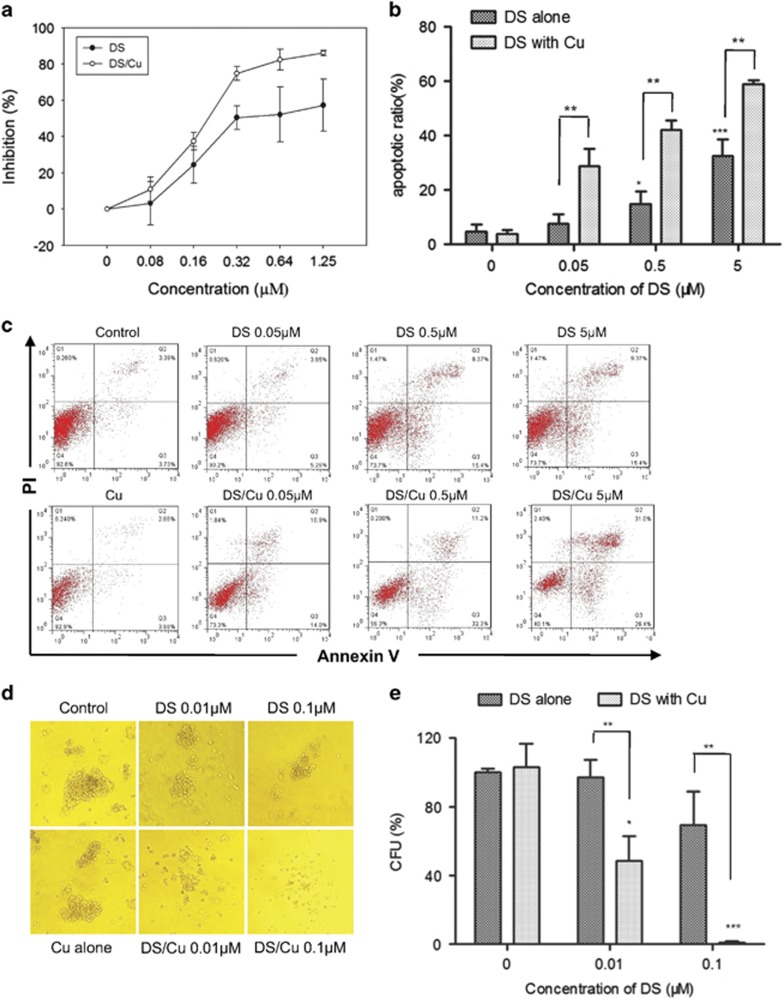
DS/Cu is cytotoxic toward leukemia stem-like cells *in vitro*. (**a**) Dose-dependent DS/Cu-induced effect on cell proliferation. (**b**) Histogram of apoptosis percentage in CD34^+^/CD38^−^ KG1a leukemia stem-like cells. **P<0.01. (**c**) Representative data for flow cytometric analysis of Annexin V/PI dual staining in CD34^+^/CD38^−^ KG1a cells after treated with indicated concentrations of DS with or without Cu (1 *μ*M) for 24 h. (**d**) Dose-dependent effect on colony-forming ability after treatment with DS or DS/Cu for 18 h. (**e**) Histogram of colony-forming ability in CD34^+^/CD38^−^ KG1a cells. *P<0.05, **P<0.01, ***P<0.001. Data were presented as mean±S.D. for three independent experiments

First, we examined the cytotoxic effect of DS/Cu on CD34^+^/CD38^−^ leukemia stem-like cells sorted from KG1*α* cells by MTT assay. As shown in Figure 2a, after exposure to a series of the indicated concentrations of DS with or without Cu (1 *μ*M) for 24 h, inhibition of CD34^+^/CD38^−^ cell proliferation was significantly increased in a dose-dependent manner, with IC_50_ value of 0.54±0.18 *μ*M for DS. Furthermore, this event was markedly enhanced by co-treatment with Cu, with a reduction of IC_50_ values to 0.21±0.03 *μ*M (DS/Cu *versus* DS, *t*=3.107, *P*=0.036), whereas treatment with Cu alone had no significant effect on cell proliferation (inhibition rate=0.66±3.36%, *P*=0.748 *versus* untreated control). Analogous results were obtained in leukemia stem-like cells sorted from Kasumi-1 cells, another human AML cell line, with 92.7±3.1% of CD34^+^/CD38^−^ cells (Supplementary Figure 1A). As shown in Supplementary Figure 1B, the inhibitory effect on cell proliferation was significantly increased after exposed to DS in combination with Cu in a dose-dependent manner, compared with DS administrated alone.

To further confirm the cytotoxicity of DS/Cu, CD34^+^/CD38^−^ KG1*α* cells were treated with DS at different concentrations (0.05, 0.5, 5 *μ*M) in the presence or absence of 1 *μ*M Cu for 24 h, and then subjected to flow cytometric analysis after Annexin V/PI double staining. Administration of Cu alone failed to induce apoptosis, compared with untreated control (5.6±4.0% *versus* 4.75±2.6%, *t*=0.474, *P*=0.66). However, co-exposure to different concentrations of DS and Cu resulted in significantly increased apoptosis in CD34^+^/CD38^−^ cells (Figures 2b and c; *P*<0.01 *versus* DS alone for each dose of DS). Similarly, in CD34^+^/CD38^−^ Kasumi-1 cells, DS in combination with Cu (1 *μ*M) also displayed a remarkably increased property of apoptosis induction in a dose-dependently manner, whereas treatment with DS alone was barely able to trigger apoptosis (Supplementary Figure 1C; DS/Cu *versus* DS alone, *P*<0.05 for each dose of DS).

We then examined whether DS/Cu would affect clonogenicity of leukemia stem-like cells, by the colony-forming assay. As shown in Figures 2d and e, whereas treatment with Cu (1 *μ*M) alone had no effect on colony formation (*P*=0.77 *versus* untreated control), exposure to DS alone moderately inhibited colony formation was (mean colony-forming units (CFU) inhibition rate, 69.29±19.54% for 0.1 *μ*M DS, *P*<0.05 *versus* untreated control), which was sharply enhanced when DS and Cu were administrated together in CD34^+^CD38^−^ KG1*α* cells (48.55±14.36% for 0.01 *μ*M DS, *P*<0.05 and *P*<0.01 *versus* untreated control and DS alone, respectively; 0.83±0.72% for 0.1 *μ*M DS, *P*<0.001 and *P*<0.01 *versus* untreated control and DS alone, respectively). Taken together, these results suggest that whereas DS itself displays noticeable dose-dependent cytotoxicity toward leukemia stem-like cells, while this effect is markedly potentiated when combined DS with non-toxic concentrations of Cu.

### DS/Cu-mediated cytotoxicity is dependent upon intracellular ROS production in leukemia stem-like cells

**Figure 3:**
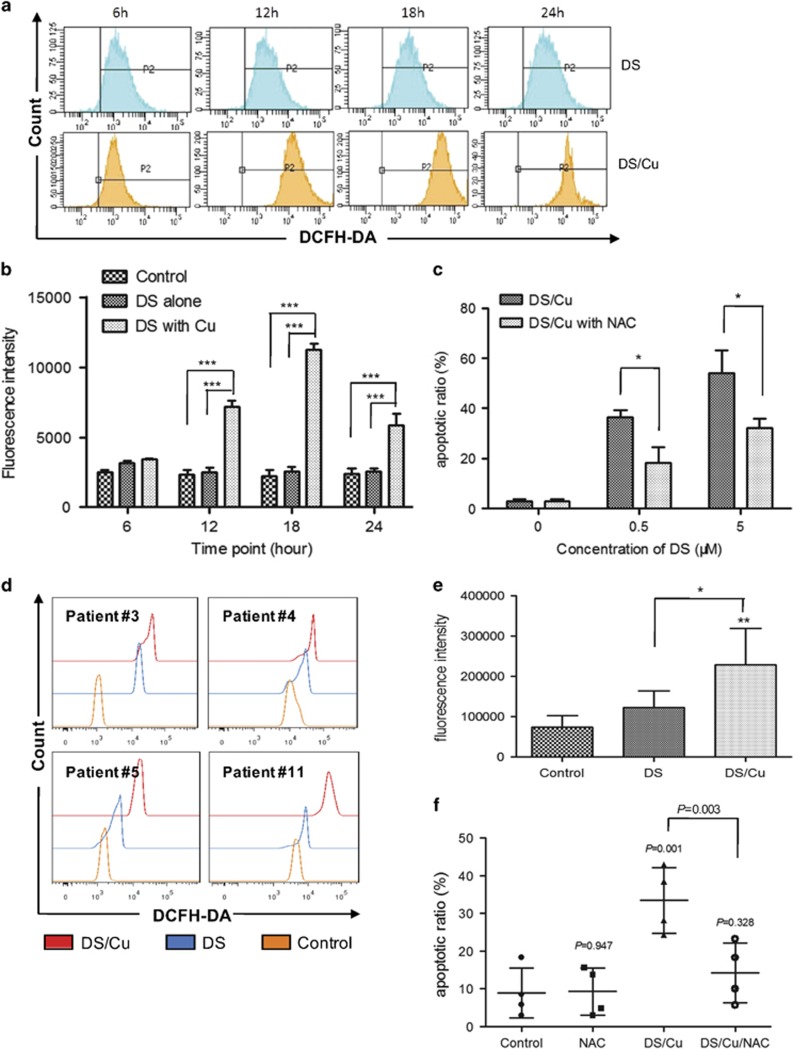
DS/Cu-induced cytotoxicity is associated with intracellular ROS accumulation in leukemia stem-like cells. (**a**) CD34^+^/CD38^−^ KG1a cells were treated with DS (0.5 *μ*M) ± Cu (1 *μ*M) for the indicated intervals. Intracellular ROS level was determined by analysis of DCFH-DA fluorescence intensity. (**b**) Histogram of ROS generation in CD34^+^/CD38^−^ KG1a. ***P<0.001. (**c**) Histogram of apoptosis percentage in CD34^+^/CD38^−^ KG1a cells in the presence of free radical scavenger NAC (10 mM) ± DS/Cu for 24 h. *P<0.05. (**d**) Intracellular ROS level was examined by analysis of DCFH-DA fluorescence intensity. Primary CD34^+^ samples isolated from AML patient #3, #4, #5, #11 were treated with DS (0.1 *μ*M) +/- Cu (1 *μ*M) for 6 h. *P<0.05, **P<0.01. (**e**) Histogram of ROS generation in CD34^+^ primary cells (*n*=4). (**f**) Histogram of apoptosis percentage in CD34^+^ primary cells (*n*=4) after treated with DS/Cu or DS/Cu/NAC

Previous studies have indicated that generation of intracellular ROS is crucial for induction of apoptosis by chemotherapeutic agents in various types of cancers.^33^ To determine whether DS/Cu-induced apoptosis would rely on intracellular ROS production, we thus measured the ROS level in CD34^+^CD38^−^ KG1*α* cells after exposed to DS/Cu for 6, 12, 18, 24 h, using the DCFH-DA-based assay. As shown in Figures 3a and b, co-administration of DS (0.5 *μ*M) and Cu (1 *μ*M) resulted in a robust increase in intracellular ROS accumulation at different time points (*P*<0.001 for each interval except 6 h, compared with untreated control and DS alone, respectively), in association with induction of apoptosis by DS/Cu (Figures 2b and c).

We next investigated the functional role of ROS in induction of apoptosis by DS/Cu in leukemia stem-like cells, by employing the free radical scavenger *N*-acetyl-cysteine (NAC). Apoptosis was analyzed after co-exposed to different concentrations of DS and Cu (1 *μ*M) in the presence or absence of NAC (10 mM) for 24 h in both CD34^+^/CD38^−^ KG1*α* and Kasumi-1 cells, respectively. As shown in Figure 3c, treatment with NAC clearly abrogated apoptosis induced by DS/Cu in CD34^+^/CD38^−^ KG1*α* cells (*P*<0.05 *versus* DS/Cu for each dose of DS), whereas NAC itself had almost no effect on apoptosis (*P*=0.762 *versus* untreated control). Moreover, identical treatment with NAC also partly rescued CD34^+^/CD38^−^ Kasumi-1 cells from apoptosis induced by DS/Cu as shown in Supplementary Figures1E and 1F (*P*<0.05 for each group, DS/Cu/NAC *versus* DS/Cu).

The similar results were also obtained in primary CD34^+^ cells isolated from bone marrow samples of the AML patients #3, #4, #5, and #11. As shown in Figures 3d–f, after exposure to DS (0.1 *μ*M) with or without Cu (1 *μ*M) for 6 h, a significant increase in intracellular ROS level was observed in primary CD34^+^ AML cells (*P*<0.01, DS/Cu *versus* untreated control; *P*<0.05, DS/Cu *versus* DS alone). Furthermore, co-administration with NAC also remarkably abolished apoptosis induced by DS/Cu in primary CD34^+^ AML cells (*P*=0.001 for DS/Cu *versus* untreated control; *P*=0.003 DS/Cu/NAC *versus* DS/Cu), whereas NAC itself did not affect apoptosis (*P*=0.947 *versus* untreated control). Together, these results suggest that DS/Cu-induced apoptosis in leukemia stem-like cells is highly dependent on generation of intracellular ROS.

### DS/Cu inhibits the Nrf2 and NF-*κ*B pathways as well as activates the JNK pathway in leukemia stem-like cells

**Figure 4:**
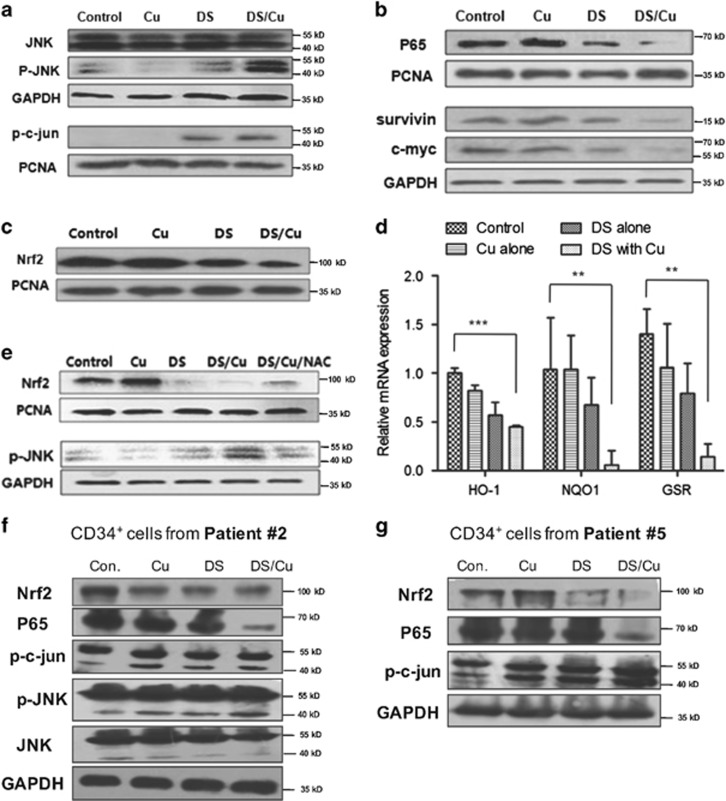
DS in combination with Cu suppresses expression of Nrf2 and NF-*κ*B while activates the JNK pathway in leukemia stem-like cells. (**a**, **b**) CD34^+^/CD38^−^ KG1*α* cells were incubated with 0.5 *μ*M DS ± 1 *μ*M Cu for 24 h, flowed by western blot analysis to monitor expression of the JNK (**a**) and NF-*κ*B (**b**) pathway-related proteins. (**c**) Western blotting analysis of Nrf2 protein level in CD34^+^/CD38^−^ KG1*α* cells after DS or DS/Cu treatment for 24 h. (**d**) Relative mRNA expression of downstream genes (HO-1, NQO1 and GSR) of the Nrf2 pathway in CD34^+^/CD38^−^ KG1*α* cells. Data were presented as mean±S.D. of three independent experiments. **P<0.01, ***P<0.001. (**e**) Western blot analysis for the expression of Nrf2 and p-JNK in CD34^+^/CD38^−^ KG1*α* cells after co-treatment with DS/Cu and NAC. (**f**, **g**) Western blot analysis for the detection of JNK, Nrf2, and P65 pathway expression in primary CD34^+^ AML cells isolated from patient #2 (**f**) and patient #5 (**g**), after exposure to DS with or without Cu for 24 h

Given the previous observations that ROS-mediated apoptosis highly relies on activation of JNK, the effect of DS/Cu on the JNK pathway was assessed in CD34^+^/CD38^−^ KG1*α* cells. As shown in Figure 4a, exposure to DS resulted in a clear increase in phosphorylation of both JNK (p-JNK) and its downstream target c-jun (p-c-jun), which was remarkably enhanced by co-administration of Cu.

Previous reports have also demonstrated that DS inhibits NF-*κ*B, in association with its potent antitumor activity.^34^ Notably, treatment with DS alone for 24 h effectively downregulated the expression of p65, a core component of the NF-*κ*B family, accompanied by markedly reduced expression of the NF-*κ*B target genes survivin and c-myc, and these events were clearly enhanced when DS was administrated with Cu in combination (Figure 4b).

As Nrf2 has an important role in activation of antioxidant responses to ROS,^8^ Nrf2 and its downstream gene expression was monitored by western blot analysis and RT-PCR after exposed to DS (0.5 *μ*M) with or without Cu (1 *μ*M) for 24 h. Treatment with DS or DS/Cu significantly reduced the protein level of Nrf2 in CD34^+^/CD38^−^ KG1*α* cells (Figure 4c). Of note, whereas DS or Cu alone displayed a modest effect (*P*>0.05 *versus* untreated control), combined administration of DS/Cu dramatically inhibited the expression of the Nrf2 downstream genes, including HO-1, NQO1, and GSR, in CD34^+^/CD38^−^ KG1*α* cells (Figure 4d).

Comparable results were also observed in primary CD34^+^ cells isolated from bone marrow samples of the AML patient #2 and patient #5. As shown in Figures 4f and g, *ex vivo* treatment with DS/Cu resulted in a sharp decrease in the protein level of p65 in both patient samples, whereas DS and/or Cu alone or in combination also increased phosphorytions of p-JNK (particularly p46 isoform) and p-c-jun (36 kDa, lower band), as well as downregulated Nrf2 in these samples in primary CD34^+^ AML cells.

### The free radical scavenger NAC reverses the DS/Cu-induced alterations in the Nrf2 and JNK pathways

To examine a possibility whether the alterations of Nrf2 expression and JNK activation would be linked to ROS, NAC (10 mM) was used to test the role of ROS in these events induced by DS/Cu. As shown in Figure 4e, downregulation of Nrf2 and phosphorylation of JNK induced by DS/Cu were markedly blocked by NAC in CD34^+^CD38^−^ KG1*α* cells, suggesting that ROS might act upstream of the Nrf2 and JNK pathways in leukemia stem-like cells treated with DS/Cu.

### DS/Cu selectively targets primary CD34^+^ AML stem-like cells while sparing normal hematopoietic progenitor cells

**Figure 5:**
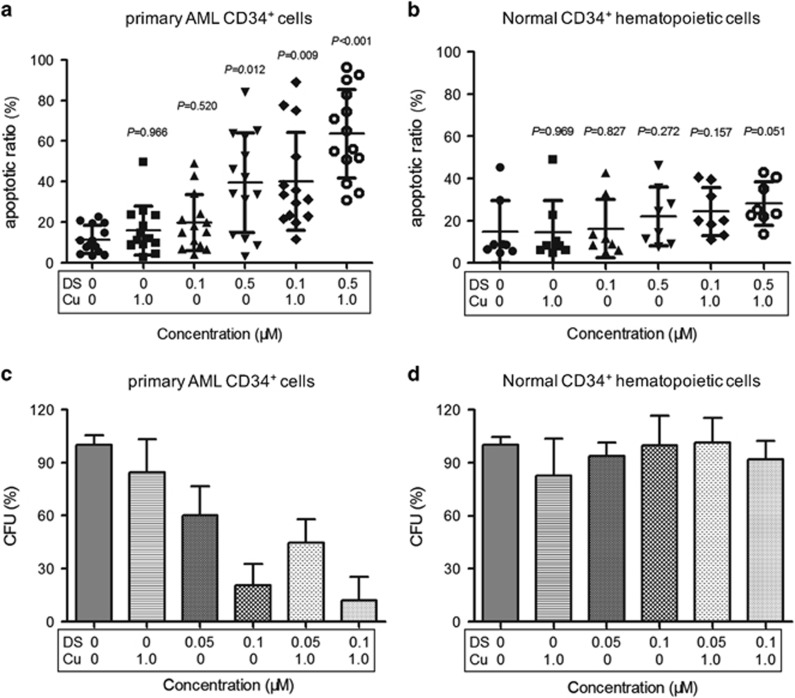
DS/Cu selectively kills primary CD34^+^ AML patients cells while sparing normal hematopoietic progenitor cells. (**a, b**) Histogram of apoptosis percentage in primary CD34^+^ AML cells (**a**) and normal CD34^+^ hematopoietic cells (**b**) after exposure to indicated concentrations of DS with 1.0 *μ*M Cu for 24 h. Each symbol represents results for an individual. Horizontal lines indicate the mean and SD for 14 AML patients and 8 healthy donors. (**c, d**) Histogram of colony-forming ability of primary CD34^+^ AML cells (**c**) and normal CD34^+^ hematopoietic cells (**d**) after treatment with DS or DS/Cu for 18 h, compared with untreated control. Data were presented as mean±S.D. for three AML patients and three healthy donors

To validate the activity of DS/Cu against leukemia stem-like cells, CD34^+^ cells were isolated by magnetic activated cell sorter (MACS) from AML patients and healthy donors for hematopoietic stem cell transplantation, respectively. Clinical characteristics of these AML patients are summarized in Table 1. Consistent with the anti-leukemia effect of DS/Cu observed in CD34^+^/CD38^−^ KG1*α* cells, treatment with 0.5 *μ*M DS with or without 1 *μ*M Cu-induced apoptosis of CD34^+^ AML cells (Figure 5a; *P*=0.012 and *P*<0.001 DS and DS/Cu *versus* untreated control, respectively; *n*=14), whereas Cu alone (1 *μ*M) was unable to induce apoptosis (15.92±12.1% *versus* 11.45±6.8% for Cu alone *versus* untreated control, *P*=0.966). Of note, although administration of DS alone at low doses (e.g., 0.1 *μ*M) did not significantly induced apoptosis (*P*=0.52, compared with untreated control), combined treatment with Cu (1 *μ*M) led to a marked increase in apoptosis of CD34^+^ AML cells (Figure 5a; *P*=0.009 for DS/Cu *versus* untreated control). In sharp contrast, the identical treatment failed to induce apoptosis in normal CD34^+^ hematopoietic cells (Figure 5b; *P*>0.05 for DS/Cu *versus* untreated control in all cases).

Three patients (#7, 8, and 12) with secondary AML (sAML) evolved from a preceding phase of myelodysplastic syndrome (MDS) were also enrolled in the present study. Unexpectedly, consistent with the *ex vivo* activity in primary samples from AML patients, DS/Cu also exhibited a potent antitumor activity against CD34^+^ cells isolated from these three patients with sAML (49.4±14.5% and 67.8±21.5% *versus* 10.6±8.3% for 0.5 *μ*M DS alone and 0.5 *μ*M DS/1 *μ*M Cu *versus* untreated control, *P*=0.002 and *P*<0.001, respectively). Further two-way analysis of variance (ANOVA) analysis revealed no significant difference in the cytotoxic effect of DS/Cu between sAML (*n*=3) and primary AML (*n*=11; *P*=0.62).

Colony formation assay was then carried out to assess clonogenicity of primary CD34^+^ AML stem-like cells and CD34^+^ normal hematopoietic cells, respectively. As shown in Figure 5c, DS alone at low doses (0.05 *μ*M and 0.1 *μ*M) significantly reduced the number of CFU of primary CD34^+^ AML cells after cultured for 10–14 days (*P*<0.05 and *P*<0.01 for 0.05 *μ*M and 0.1 *μ*M *versus* untreated control, respectively), whereas combined treatment with DS and Cu (1 *μ*M) further reduced the number of CFU (*P*<0.01 *versus* untreated control in all cases). It is noteworthy that the identical treatment with DS alone or DS/Cu exhibited almost no effect on the number of CFU of normal CD34^+^ hematopoietic cells (Figure 5d; *P*>0.05 *versus* untreated control for each condition). Therefore, these findings suggested that DS/Cu might have highly effective anti-leukemia activity against leukemia stem-like cells, with low toxicity toward normal hematopoietic progenitor cells.

### DS/Cu suppresses growth of xenograft derived from CD34^+^/CD38^−^ leukemia stem-like cells

**Figure 6:**
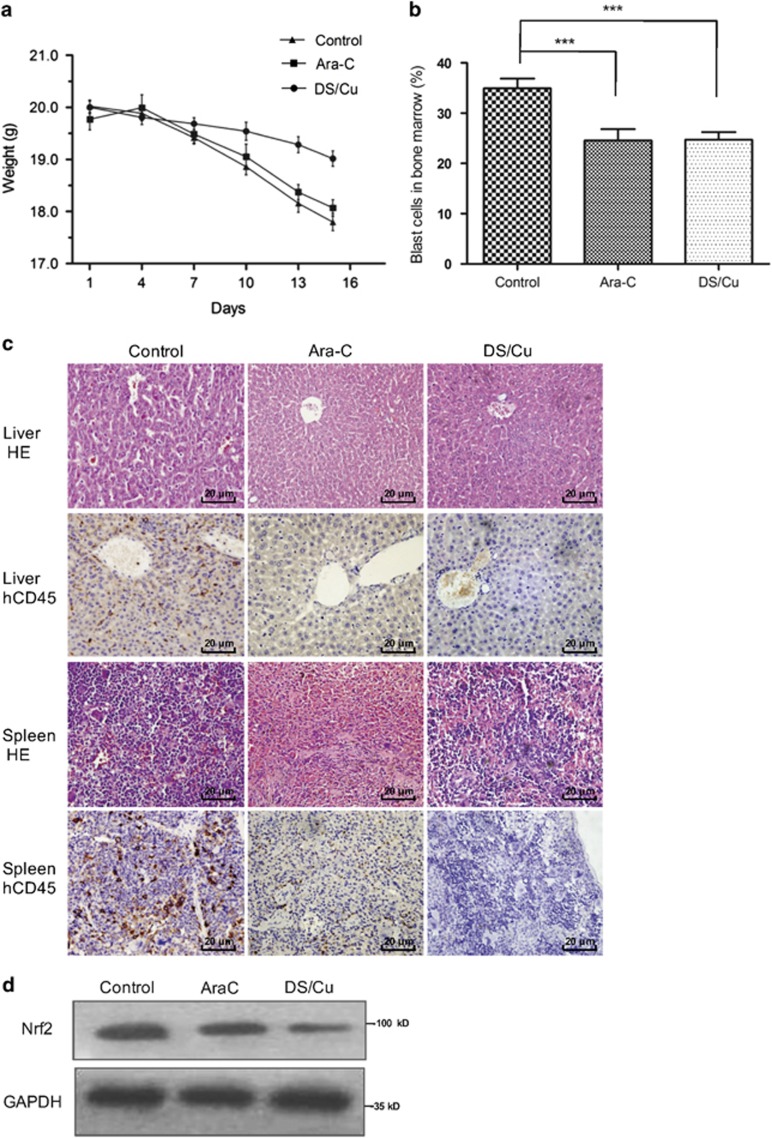
DS/Cu suppresses growth of xenograft derived from leukemia stem-like cells. An xenograft model was established in NOD/SCID mice 4 weeks after inoculation of CD34^+^/CD38^−^ KG1a leukemia stem-like cells. Mice were randomly divided into DS/Cu group, Ara-C group and control group (eight per group). (**a**) Body weight changes of mice received different treatments. (**b**) Histogram of proportion of blast cells in bone marrow evaluated microscopically after Wright staining. ***P<0.001. (**c**) Paraffin-embedded sections of spleen and liver from mice were subjected to H&E staining and immunohistochemcal staining for hCD45 (× 400). (**d**) Western blot analysis of spleen extracts from mice using anti-Nrf2 antibody

Last, to assess *in vivo* antitumor effects of DS/Cu, CD34^+^/CD38^−^ KG1*α* cells were grafted in mice to a leukemia mouse model as described in detail in Methods. As shown in Figure 6a, a significant inhibition of tumor growth was observed after DS/Cu treatment, arguing the antitumor activity of DS/Cu *in vivo*. Average body weight of mice treated with vehicle (PBS) was 17.80±0.41 g after 2 weeks, whereas was 18.07±0.35 g and 19.02±0.39 g for Ara-C group and DS/Cu group, respectively (*P*<0.05 for DS/Cu group *versus* the group of mice received either vehicle or Ara-C, a standard agent currently used to treat AML in clinic). In addition, treatment with either DS/Cu and Ara-C remarkably reduced tumor burden, manifested by a significant decrease in percentage of blast cells in bone marrow (Figure 6b; 24.73±1.48% for DS/Cu group *versus* 34.96±1.94% for vehicle group, *P*<0.001; 24.56±2.25% for Ara-C group *versus* 34.96±1.94% for vehicle group, *P*<0.001). However, there was no statistically significant difference between DS/Cu and Ara-C groups (*P*>0.05). Moreover, HE staining and immunohistochemistry for human CD45 at 6th week following transplantation demonstrated that the ability of blast cells to invade liver and spleen was remarkably impaired in mice treated with DS/Cu or Ara-C, compared with those in mice received vehicle as control (Figure 6c). Finally, western blot analysis revealed that treatment with DS/Cu also reduced Nrf2 protein level in spleen cells (Figure 6d), consistent with the results observed *in vitro*.

## Discussion

Development of new drug is a costly and time-consuming process. Therefore, exploring new functions for existing drugs, termed repurposing or repositioning, has attracted great attention owing to low cost and fast track. DS, a member of the dithiocarbamate family and which is an aldehyde dehydrogenase inhibitor and a Cu chelator, has been approved by FDA for the treatment of alcoholism for more than six decades.^9, 10^ Cu is an essential trace element for tumor cell proliferation and thus could serve as a selective target for cancer therapies.^22^ Despite the concept of using Cu to tackle cancer that was proposed many decades ago, intracellular transport of Cu remains a major hurdle for its clinical application. However, this problem might be solved by combination of Cu with DS, as the derivative *N*,*N*-diethyldithiocarbamte (deDTC) of DS can bind to Cu to form a Cu(deDTC)_2_ complex and thereby improve the intracellular trafficking of Cu.^13^

Recently, the anticancer activity of DS have attracted many attentions in various types of cancers. Here, we demonstrated that DS, particularly when co-administrated with non-toxic concentrations of Cu (e.g., 1 *μ*M), was highly cytotoxic to leukemia stem-like cells, including CD34^+^/CD38^−^ KG1*α* cells and CD34^+^/CD38^−^ Kasumi-1, as well as primary CD34^+^ cells isolated from AML patients, in a dose-dependent manner, whereas largely sparing normal hematopoietic progenitor cells. Compared with normal cells, AML cells might generally have higher level of Cu, which enables DS to target AML cells selectively. The present *in vitro* results indicate that in the absence of Cu, the concentration of DS required for antitumor action was markedly higher than that in the presence of Cu (0.54 *μ*M *versus* 0.21 *μ*M) in CD34^+^CD38^−^ KG1*α* cells, suggesting the necessity of free Cu in the cytotoxic effect of DS/Cu in combination. Of note, IC_50_ of DS in combination with non-toxic Cu against leukemia stem-like cells was significantly lower than the concentration recommended for alcoholism treatment (500 mg as maximum daily dosage),^35^ implying high antitumor efficacy but low non-specific toxicity of DS. In addition to the anti-proliferation function, DS/Cu also had a marked effect on both induction of apoptosis and impairment of clonogenicity of leukemia stem-like cells, including primary CD34^+^ AML cells, with minimally toxicity towards normal counterparts obtained from healthy donors. We also obtained encouraging results in xenograft animal models derived from leukemia stem-like cells, reflected by both reduction of tumor burden as well as inhibition of blast cell invasion into vital organs such as liver and spleen. Together, these findings argue that DS/Cu has a promising potential to be safely used to treat AML in clinic.

sAML is a poorly defined term that often refers to AML developed following a previous disease, such as MDS or chronic myeloproliferative disorder.^36^ Approximately 30% of patients with MDS will develop to AML, especially those with high-risk MDS. Poor clinical outcomes for this large and variable group of patients with sAML are associated with greater incidence of chemo-resistance compared with those with *de novo* AML. Interestingly, the marked *ex vivo* activity of DS/Cu was observed in CD34^+^ cells isolated from patients with sAML developed from MDS, without any significant difference compared with those from patients with primary AML, suggesting that patients with sAML might be also susceptible to DS/Cu treatment. However, although the role of DS/Cu in patients with sAML remains to be defined in a larger sample size of study, this finding provides initial evidence for further exploration of DS/Cu in treatment of sAML evolved from MDS.

It is well characterized that antitumor activity of most chemotherapeutic agents is closely related to both generation of ROS and disruption of redox homeostasis. As a consequence, ROS induces collapse of mitochondria membrane potential, thereby triggering a series of mitochondria-associated events including apoptosis.^37^ In consistence with these results, measurement of ROS level in leukemia stem-like cells indicated that exposure to DS/Cu led to a marked intracellular ROS burst, which functionally contribute to DS/Cu-induced apoptosis, manifested by the abrogation of cell death by the antioxidant NAC. Moreover, it has been reported that ROS is a potent activator of JNK through oxidative inactivation of endogenous JNK inhibitors, such as JNK phosphatases and glutathione S-transferase *π*.^19^ The results of the present study indicated that sustained activation of the JNK pathway (e.g., phosphorylation of JNK and c-Jun) was induced by DS/Cu in leukemia stem-like cells while reversed by NAC that inhibited ROS production, supporting a notion that ROS-activated JNK signal might be, at least in part, responsible for DS/Cu-induced apoptosis.

It has widely accepted that in addition to induction of ROS, the effect of anti-neoplastic agents is also associated with their capability to inhibit activity of pro-survival transcription factors and antioxidants. NF-*κ*B is known to be aberrantly activated in several types of tumor cells, including LSCs.^38^ Constitutive activation of NF-*κ*B may provide an unique opportunity to preferentially target LSCs. Inhibition of NF-*κ*B can also induce intracellular ROS generation, leading to cell death.^14^ Consistent with previously observations that DS is an effective inhibitor of NF-*κ*B, DS indeed downregulated p65 and inhibited expression of the NF-*κ*B downstream genes in leukemia stem-like cells, whereas this event was markedly enhanced by co-administration of Cu. Activation of the Nrf2 signaling pathway represents a major mechanism for cellular defense against oxidative stress. Its downstream targets are involved in detoxification of harmful chemicals and removal of reactive oxidants, consequently protecting cells from detrimental stresses.^28^ Therefore, Nrf2 might be a potential target for elimination of LSCs. In the present study, we observed that DS and DS/Cu sharply reduced the protein level of Nrf2 both *in vitro* and *in vivo* in leukemia stem-like cells, as well as blocked the expression of Nrf2 target genes such as NQO1, GSR, and HO-1. Downregulation of Nrf2 was partially reversed by NAC, indicating that ROS increase might result in decrease of Nrf2, thereby impairing its ability to protect cells from ROS-mediated oxidative damage. Taken together, in addition to activation of the ROS-JNK signaling cascade, inactivation of NF-*κ*B and Nrf2 might also contribute to anti-leukemia activity of DS/Cu toward leukemia stem-like cells. It is noteworthy that these pro-survival signaling pathways (e.g., NF-*κ*B and Nrf2), as well as the stress-related ROS-JNK pathway, are determinants for tumor cell survival *versus* death in a variety of cancers.^39, 40, 41, 42, 43, 44, 45^ Thus, inactivation of NF-κB and Nrf2 signals and/or activation of the ROS-JNK signaling cascade might represent common mechanisms of action underlying the antitumor activity of DS/Cu in diverse cancers, including hematologic malignancies (e.g., myeloid and lymphoid leukemias) as we observed earlier.^19, 46^

Collectively, the ability of DS in combination with Cu to eliminate leukemia stem-like cells *in vitro* and *in vivo* indicate that DS/Cu might represent a promising therapeutic repurposed agent to preferentially target LSCs, while sparing normal hematopoietic progenitor cells. Mechanistically, the anti-leukemia property of DS/Cu might attribute to induction of apoptosis via ROS-mediated activation of the stress-related JNK pathway and inactivation of the NF-*κ*B and Nrf2 pathways. Therefore, the combination of DS and Cu warrants further clinical investigation in treatment of refractory leukemia, particularly targeting LSCs to reduce recurrence of this disease.

## Materials and Methods

### Reagents

DS, copper chloride (CuCl_2_), and NAC were purchased from Sigma-Aldrich (Dorset, UK) and dissolved in dimethyl sulfoxide or PBS, and freshly diluted to required concentrations in subsequent experiments with culture medium before use.

### Primary patient samples

Mononuclear cells were isolated from peripheral blood samples of healthy donors for hematopoietic stem cell transplantation (*n*=8) and bone marrow samples of newly diagnosed patients with AML (*n*=17), which were obtained with the informed consent for research purposes only at Department of Hematology, Nanfang Hospital, Southern Medical University and Department of Hematology, First Affiliated Hospital of Xiamen University. This study is approved by the Ethics Review Board of Nanfang Hospital and First Affiliated Hospital of Xiamen University, and performed in accordance with the Declaration of Helsinki. Clinical characteristics are summarized in Table 1. Mononuclear cells were isolated by density gradient centrifugation using Lymphoprep (BD, Franklin Lakes, NJ, USA) and cultured in IMDM (HyClone, Thermo Scientific, Waltham, MA, USA) supplemented with 10% fetal bovine serum (FBS, Gibco, Life Technologies, Grand Island, NY, USA), 100 U/ml penicillin and 100 *μ*g/ml streptomycin (1 × *P*/S) after enrichment with a CD34 selection MACS kit (Miltenyi Biotec, Bergisch Gladbach, Germany) according to the manufacturer's protocol.

### Cell culture and sorting

KG1*α* and Kasumi-1 cells were obtained from Tianjing Institute of Hematology, Chinese Academy of Medical Sciences and cultured in IMDM supplemented with 10% FBS and antibiotics (1 × *P*/S) in a 37 °C incubator with 5% CO_2_. The CD34^+^/CD38^−^ population was obtained by staining with CD34-allophycocyanin (CD34–APC; Becton Dickinson Biosciences, Oxford, UK) and CD38-fluorescein isothiocyanate (CD38–FITC; Becton Dickinson Biosciences) and then subjected to sorting using FACS Aria II.

### Cell viability assay

Cell viability was determined by MTT assay. CD34^+^/CD38^−^ KG1*α* cells and CD34^+^/CD38^−^ Kasumi-1 cells were inoculated in a 96-well plate at a density of 2 × 10^4^/well in 100 *μ*l growth medium and then treated with designated doses of DS with or without Cu (1 *μ*M) for 24 h. Then, a final concentration of 5 mg/ml MTT in PBS was added to each well and incubated for 4 h. Viable cells were detected by measuring absorbance at 490 nm using an Multisckan MCC340 microplate reader (Labsystem, Helsinki, Finland). All experiments were conducted in triplicate. IC_50_ value (half maximal inhibitory concentration) was calculated by GraphPad Prism 5.

### Flow cytometric analysis of apoptosis

Annexin V-FITC/PI staining was performed according to the manufacturer’s instruction to detect the apoptotic cells. In brief, CD34^+^/CD38^−^ KG1*α* cells, CD34^+^CD38^−^ Kasumi-1 cells, primary CD34^+^ AML cells, and normal CD34^+^ hematopoietic cells were treated with escalating concentration of DS with or without Cu (1 *μ*M) for 24 h. Cells were harvested after washing twice with iced PBS and then stained with Annexin V-FITC/PI for 20 min at 4 °C in the dark. The percentage of apoptotic cells was determined by FACS C6 (BD, Oxford, UK). At least 30 000 gated events were acquired from each sample.

### Measurement of intracellular ROS

Cells were exposed to DS alone or in combination with Cu (1 *μ*M) for indicated time points (6, 12, 18, 24 h), then supernatants were removed and cells were labeled with 2′,7′-dichlorofluorescin-diacetate (DCFH-DA, Invitrogen, Paisley, UK) at 37 °C for 30 min. Cells were washed twice with PBS and maintained in 1 ml serum-free medium. Cellular ROS level was analyzed using a FACS Calibur machine (Becton Dickinson, San Jose, CA, USA). Each sample was measured at least in triplicate.

### Colony formation assay

The colony-forming capacity of CD34^+^/CD38^−^ KG1*α* cells and CD34^+^ primary AML cells was measured using methylcellulose medium (Methocult H4434, Stem Cell Technologies, Vancouver, BC, Canada) according to the manufacturer's instructions. In brief, cells were cultured in six-well plates with Methocult H4434 at a density of 500 cells/well after treated with DS alone or in combination with Cu (1 *μ*M) for 18 h. After incubation at 37 °C for 10–14 days, CFU consisting of 40 or more cells were counted under microscope.

### Western blot analysis

After cell lysis, 50 *μ*g/lane of proteins from each sample was resolved in 10% SDS-polyacrylamide gel electrophoresis and then transferred to PVDF membrane (Millipore, Billerica, MA, USA). After blocking with 5% non-fat milk in Tris-buffered saline-Tween 20 (TBS-T) for 2 h, the membrane was incubated overnight at 4 °C with the primary antibody, followed by appropriate secondary HRP-conjugated antibody (1:10 000, Cell Signaling Technology, Herts, UK) in TBS-T for 2 h at room temperature. The proteins were visualized on X-ray film using an ECL Western blotting detection kit (Cell Signaling Technology). GAPDH and PCNA were probed as loading controls.

### Quantitative real-time PCR (qPCR)

Cells were lysed with Trizol reagent (Invitrogen, Carlsbad, CA, USA) and total RNA from each sample was extracted with chloroform and isopropyl alcohol. cDNA was synthesize using a reverse transcription reagent kit (TaKaRa, Dalian, China) according to the manufacturer’s instructions. Quantitative real-time PCR was carried out by a SYBR Prime Script RT-PCR kit (Takara) using the following primers (synthesized by Takara). The relative levels of mRNA expression were determined by the 2^−ΔΔCt^ method.

NQO1: 5′-GTGGCAGTGGCTCCATGTACTC-3′(forward),

5′-GAGTGTGCCCAATGCTATATGTCAG-3′ (reverse);

GSR: 5′-CCTGATCGCCACAGGTGGTA-3′ (forward),

5′- CTGCCATCTCCACAGCAATGTAA-3′ (reverse);

HO-1: 5′- TTGCCAGTGCCACCAAGTTC-3′ (forward),

5′- TCAGCAGCTCCTGCAACTCC-3′ (reverse);

GAPDH: 5′-TCTCTGCTCCTCCTGTTC-3′(forward),

5′-CTCCGACCTTCADCTTCC-3′ (reverse);

### Animal study

NOD/SCID mice (6–8 weeks old, non-fertile, female, and 18–20 g each) were purchased from the Experimental Center of Southern Medical University and bred in a pathogen-free environment and supplied with sterile food and water. All animal study procedures were approved by Nanfang Hospital of Southern Medical University Animal Care and Use Committee. After receiving 1 Gy of sublethal irradiation, mice were intravenously injected with 2  ×  10^6^ CD34^+^/CD38^−^ KG1*α* cells resuspended in 200 *μ*l PBS and then monitored weekly for signs of weight loss or lethargy, and leukocytes in peripheral blood. After percentage of hCD45^+^ in peripheral blood reached 1%, mice were randomly assigned to three groups (*n*=8 per group). DS/Cu treatment was given by gavage as follows: 3 mg/20 g/day for DS per morning and 0.03 mg/20 g/day for Cu per afternoon for 2 weeks. Ara-C treatment was given as follows for comparison: 40 mg/20 g/day for 2 days before killing. Mice of the control group received vehicle (i.e., PBS, 0.2 ml/20 g/d for 2 weeks). Mice were killed after 2 weeks and invasion of leukemia cells was evaluated by H&E staining for pathological changes and immunohistochemical staining for hCD45.

### Immunohistochemical staining

Paraffin-embedded liver and spleen tissue from mice were sectioned and deparaffinized with xylene. Then slides were subjected to rehydration with different concentrations of alcohol. For antigen retrieval, slides were immersed in boiling citrate buffer for 30 min and then incubated with primary antibody against hCD45 overnight at 4 °C after blocking with 3% BSA solution at room temperature for 30 min. After washing, HRP Polymer Conjugate Reagent (SuperPicture Polymer Detection kit) and DAB Chromogen were added. Mayer’s Hematoxylin solution was used for counterstaining. Finally, slides were dehydrated, air-dried and mounted. For hematoxylin and eosin staining (H&E stain) slides were immersed in a coplin jar containing Mayer’s hematoxylin solution, washed with distilled water and then counterstained with eosin Y solution.

### Statistical analysis

All statistical analyses were performed using the SPSS 13.0 statistical software package. Comparisons between two groups were analyzed using the two-tailed Student’s *t*-test. Comparisons among multiple groups were performed using the one-way ANOVA, followed by LSD test if the variance was homogenous, whereas if not, Dunnett’s T3 was employed.*P<0.05 was considered as statistical significance.

## Figures and Tables

**Table 1:** Characteristics of patient samples

**Patients no.**	**Age (years)**	**Gender**	**FAB subtype**	**Cell count (× 10**^**9**^**/l)**			**Blast (%)**		**LDH value (U/l)**	**Extramedullary infiltration**	**Karyotype**	**Molecular features**
				**WBC**	**HB**	**PLT**	**PB**	**BM**				
1	38	M	M1	78.53	117	168	90	90.5	229.9	N	NA	–
2^a^	58	F	M4	14.49	106	64	26	44.5	551.6	N	46,XX; del(11)(q23)	–
3^b^	41	M	M2	51.35	87	26	88	89	104	N	NA	P53
4^b^	49	F	M5	75.85	103	40	72	77.5	923.6	N	46, XX	NMP1
5b,^a^	52	F	M2	29.43	95	52	46	61.5	1714	N	46,XX,t(8,21)(q22;q22)	AML1/ETO
6	57	M	M5	83.37	60	60	47	60.5	540	N	46, XY	–
7^c^	57	F	MDS/AML	2.03	74	42	23	30	126	N	46, XX	AML1/ETO; IDH2; RUNX1; SRSF2
8^c^	66	F	MDS/AML	127.68	61	58	46	70.5	787	Y	46, XX	P53; ASXL1; NOTCH1; RNRAS; RUNX1;PTPN11
9	23	M	M2	92.24	136	66	84	68.5	731.8	Y	46, XY	–
10	59	F	M2	31.43	76	96	70	69	1416	N	46, XX	–
11^b^	27	F	M2	4.33	89	15	24	38.5	247	N	46,XX,t(8,21)(q22;q22)	AML1/ETO
12^c^	58	M	MDS/AML	94.81	53	57	44	36	550	Y	46, XY	DNMT3A; RUNX1;SRSF2
13	66	M	M5	328.76	46	35	81	87.5	1096	NA	NA	NA
14	34	F	M1	15.5	66	20	78	84	358	Y	NA	–
15^d^	26	M	M2	25.32	146	122	84	73.5	357	Y	47, XY, +8	EVI1
16^d^	52	M	M5	97.3	72	108	57	61.5	140	N	46, XY	HOX11; NPM1
17^d^	47	F	M2	12.81	64	315	40	26.5	153.3	N	46, XX	AML1/ETO

Abbreviations: BM, bone marrow; F, female; FAB, French–American–British; HB, hemoglobin; LDH, lactic dehydrogenase; M, male; PB, peripheral blood; PLT, platelet; WBC, white blood cell.

a Samples used for western blotting.

b Samples used for detection of ROS.

c Patients with secondary acute myeloid leukemia (sAML) derived from myelodysplastic syndromes (MDS).

d Samples used for detection of clonogenicity ability.
