# Supplementary figures and images for: Disulfiram/copper selectively eradicates AML leukemia stem cells in vitro and in vivo by simultaneous induction of ROS-JNK and inhibition of NF-κB and Nrf2
Source: Cell Death Dis. 2017 May 18;8(5):e2797–. doi: 10.1038/cddis.2017.176 (PMC5520701; doi:10.1038/cddis.2017.176)

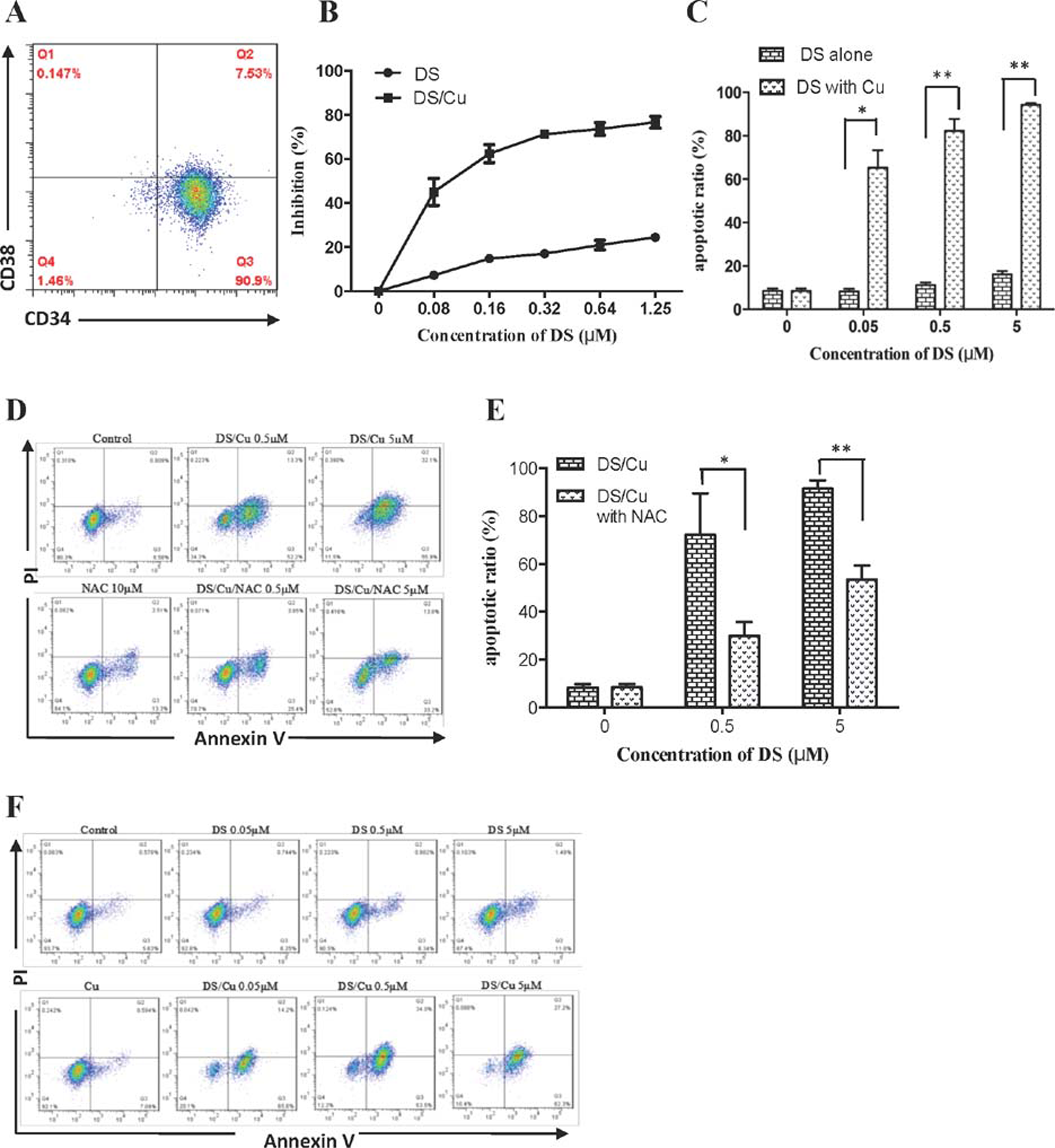

Supplement: Supplementary Figure S1 [file cddis2017176x1.tif]
