# Supplementary material for: Disulfiram/copper selectively eradicates AML leukemia stem cells in vitro and in vivo by simultaneous induction of ROS-JNK and inhibition of NF-κB and Nrf2
Source: Cell Death Dis. 2017 May 18;8(5):e2797–. doi: 10.1038/cddis.2017.176 (PMC5520701; doi:10.1038/cddis.2017.176)
Supplement: Supplementary Figure Legend [file cddis2017176x2.docx]

**Supplemental Figure 1. DS/Cu is cytotoxic to leukemia stem-like cells sorted from Kasumi-1 cell line.** (**A**) CD34 and CD38 expression in Kasumi-1 cells. (**B**) Dose-dependent DS/Cu-mediated effect on proliferation of CD34^+^/CD38^-^ Kasumi-1 cells. (**C**) Histogram of apoptosis percentage in CD34^+^/CD38^-^ Kasumi-1 cells. *P<0.05, **P<0.01. (**D**) Representative data for flow cytometric analysis of Annexin V/PI staining in CD34^+^/CD38^-^ Kasumi-1 cells after exposed to indicated concentration of DS with or without Cu (1 μM) for 24 hrs. (**E**) Histogram of apoptosis percentage in CD34^+^/CD38^-^ Kasumi-1 cells after co-treatment with DS/Cu +/- NAC (10 mM). *P<0.05, **P<0.01. (**F**) Representative data for for flow cytometric analysis of Annexin V/PI staining in CD34^+^/CD38^-^ Kasumi-1 cells after incubated with DS/Cu +/- NAC for 24 hrs. Data was presented as mean ± S.D. for three independent experiments.
